# Clinical experience and treatment strategy of leiomyosarcoma originating from the renal vein

**DOI:** 10.1186/s40001-022-00721-z

**Published:** 2022-06-27

**Authors:** Qais Baheen, Hai Bi, Kai Wang, Min Lu, Hongxian Zhang, Lulin Ma

**Affiliations:** 1 Department of Urology, Peking University Third Hospital, Haidian District, Beijing, 100191 People’s Republic of China; 2 Department of Pathology, Peking University Third Hospital, Haidian District, Beijing, 100191 People’s Republic of China

## Abstract

**Background:**

Leiomyosarcoma originating from the renal vein (RVLMS) is extremely rare. RVLMS lacks specific clinical manifestations and specific imaging features. This article discusses the epidemiological characteristics and diagnostic difficulties of RVLMS, as well as imaging features, differential diagnosis, treatment strategy, and prognostic factors of this disease.

**Method:**

A case of RVLMS at our center, and 55 cases from the literature based on the PubMed search.

**Results:**

Total operation time was 224 min, and total blood loss during the surgery was 200 ml. Resected tumor was irregular in shape, with negative margins. On the 6th day after the operation, the drainage tube was removed, and the patient was discharged from the hospital. Postoperative pathological results confirmed the renal vein leiomyosarcoma: spindle cell sarcoma, diffuse severe atypia, S-100 (-), SMA ( +), desmin ( +), CD34 (−), CD99 ( +).  Twenty-seven months after the surgery, the patient is alive, and without local recurrence or distant metastases.

**Conclusion:**

Unspecific clinical manifestations and imaging features make the diagnosis of RVLMS difficult. Most patients are diagnosed intra-operatively or following postoperative pathology. Differential diagnosis with paraganglioma (PG) and retroperitoneal sarcoma (RPS) should be made. Early and complete resection is considered as the first choice of treatment, and whether to preserve the kidney is based on the patient's condition. RVLMS is highly malignant, and may recur locally or metastasize to distant locations; therefore, adjuvant therapy and regular follow-up should be carried out after surgery.

## Background

Angiogenic leiomyosarcoma is a rare soft tissue sarcoma, which mostly occurs in the inferior vena cava. A leiomyosarcoma arising from the renal vein is even more rare. To date, only 67 cases are reported in literature [1, 2]. Similar to leiomyosarcoma arising from the inferior vena cava, renal vein leiomyosarcoma (RVLMS) lacks specific clinical manifestations. Most patients are asymptomatic, and the tumor is discovered incidentally. Symptoms such as upper abdominal pain or sore back pain can be more apparent when the tumor becomes big enough to invade surrounding tissues or when the tumor blocks the renal vein re-flux. The features of RVLMS on imaging studies are similar to those of vascular invasion by retroperitoneal tumors or renal cell carcinoma (RCC) [3]. Therefore, preoperative diagnosis is very difficult, and most patients are diagnosed by intra-operative exploration and postoperative pathology. A patient with unilateral RVLMS was admitted to our hospital in December 2019. In this article, we share the process of diagnosing the tumor, the management strategy for this patient, as well as a literature review, in the hope of providing further reference for the diagnosis and treatment of this disease.

## Method

### Patient information

**Fig. 1:**
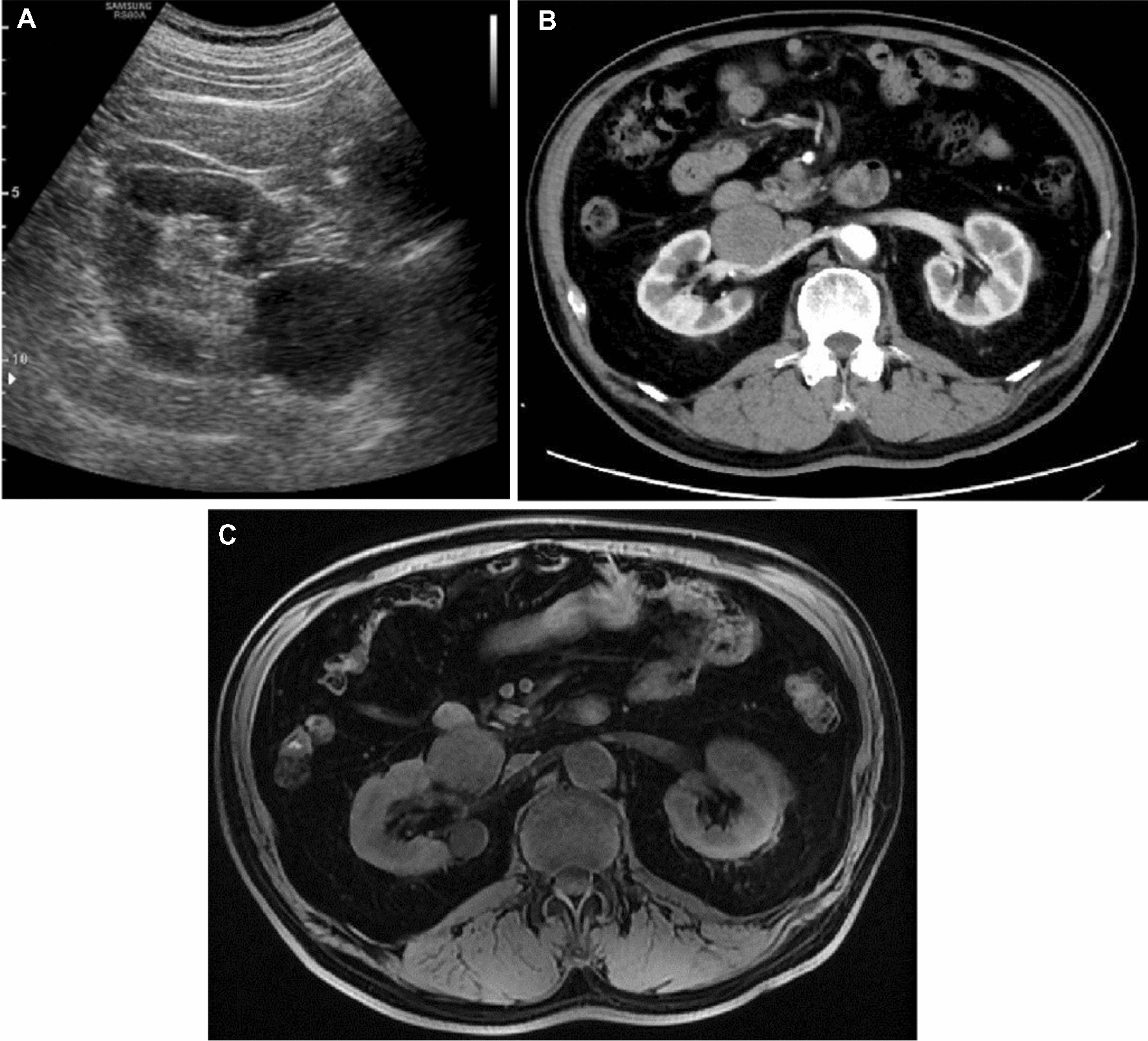
Preoperative abdominal ultrasonography (**A**), abdominal CT (**B**), and abdominal MRI (**C**)

A 61-year-old Chinese man with a history of grade 1 hypertension complained of right lower abdominal distention and intermittent pain in the upper abdomen for 4 months. Preoperative abdominal ultrasonography, computed tomography (CT), magnetic resonance imaging (MRI), serum creatinine (SCr), 24-h 3-methyl-4-hydroxymandelic acid (VMA), adrenocorticotropic hormone (ACTH), sex hormone screening, plasma cortisol rhythm and renin–angiotensin II–aldosterone system (RAAS) function in the upright and recumbent positions were performed. The preoperative contrast-enhanced ultrasonography, abdomen CT scan and MRI images are shown in Fig. 1a-c. Ultrasonography showed a 4.4 cm × 3.4 cm hypoechoic nodule with clear boundaries and internal cord-like hyperechoic mass in the right hilum (Fig. 1a). Contrast-enhanced CT scan showed a round soft tissue nodule shadow in the right renal hilum with clear boundaries, uneven enhancement and a filling defect in the renal vein cavity (Fig. 1b). MRI showed a 4.2 × 3.2 × 3.5 cm mass with clear boundaries, slightly high DWI signal and significantly low ADC value (Fig. 1c). We gave the patient phenoxybenzamine 1 month before the surgery, as diagnosis of paraganglioma (PG) was not ruled out.

### Surgical procedure

**Fig. 2:**
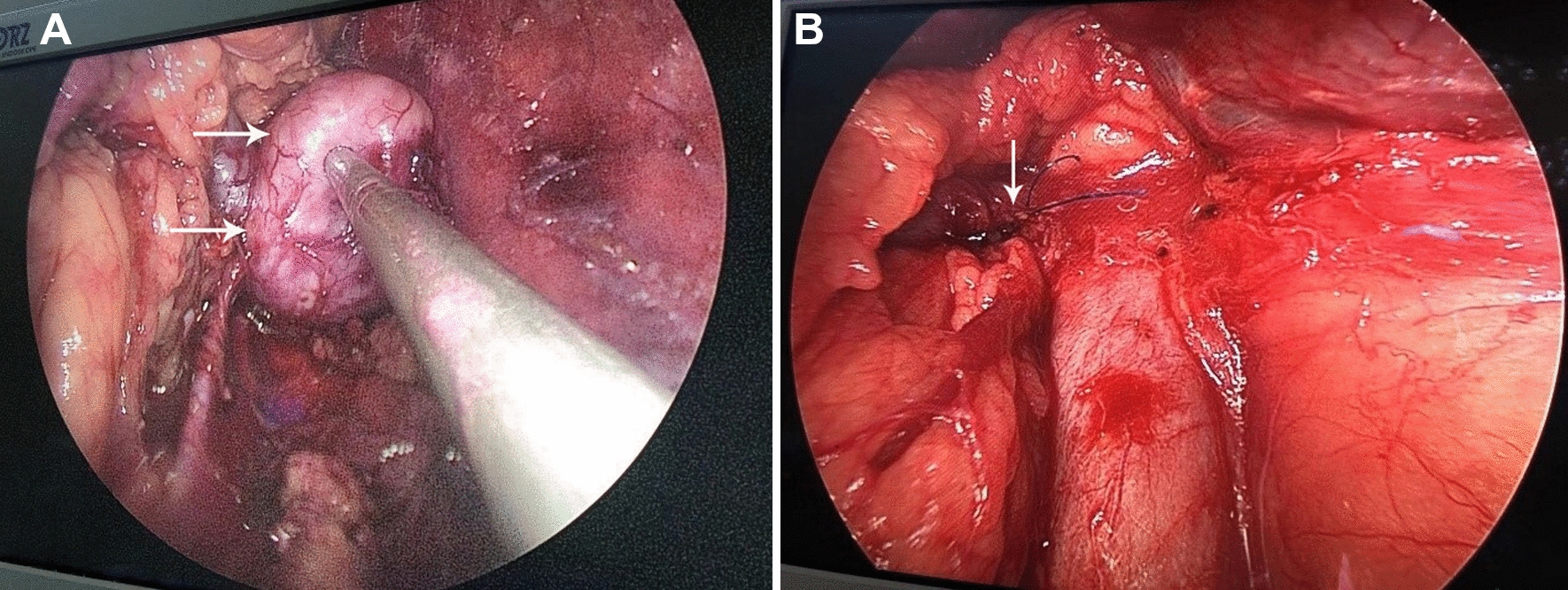
Intra-operative exploration   shows that the tumor is located very close to the right kidney (**A**), vascular suture was performed after the local excision of RVLMS

Retroperitoneal laparoscopic surgery was performed under general anesthesia. The patient was placed in lateral position, and the pneumoperitoneum was established routinely. 12 mm, 12 mm and 11 mm trocars were placed under the 12th costal margin along the right psoas major muscle, under the anterior costal margin along the axillary line, and on the iliac crest along the middle axillary line, respectively. At the same time, 5-mm trocar was placed 5 cm above the anterior superior iliac spine. We dissected the posterior layer of the Gerota fascia on the dorsal side of the kidney to the hilum of the kidney along the surface of psoas major muscle, then cut off the lymphatic vessels around the renal pedicle, and then dissected one renal artery and one renal vein. At the same time, we carefully dissected the ureter and the vena cava located near the lower pole of the kidney. We found the retroperitoneal mass is located on the dorsal side of vena cava and renal vein. After completely freeing the lower pole, ventral and dorsal sides of the kidney, we lifted the kidney, and freed the tumor on the ventral side at the angle of intersection of the right renal vein and vena cava. It was found that the tumor was irregular in shape, and had clear boundaries with the surrounding tissues. The tumor originated from the wall of the renal vein and partially protruded into the right renal vein cavity (Fig. 2a). Because the tumor was very close to the right kidney, and there was a high risk of bleeding during resection, we converted to open surgery and preserved the right kidney. We made the incision about 20 cm below the 12th costal margin, and then dissected the perirenal vessels. We blocked the right renal artery first, then partially blocked the vena cava with a bulldog clamp, and then blocked the proximal renal vein with Pug forceps. Afterwards, we opened the renal vein, and completely removed the tumor, and then reconstructed the blood vessel using a 4-0 Prolene vascular suture (Fig. 2b).

### Follow-up

After discharge from the hospital, the patients underwent follow-up every 3 months in the first year, and every 6 months afterwards. Each follow-up required clinical examination and imaging studies. In the first year, abdominal CT scans, along with routine blood tests, liver and kidney function tests were conducted. Starting from the second year, abdominal ultrasonography examinations along with routine blood tests, liver and kidney function tests were conducted.

## Results and pathology

**Fig. 3:**
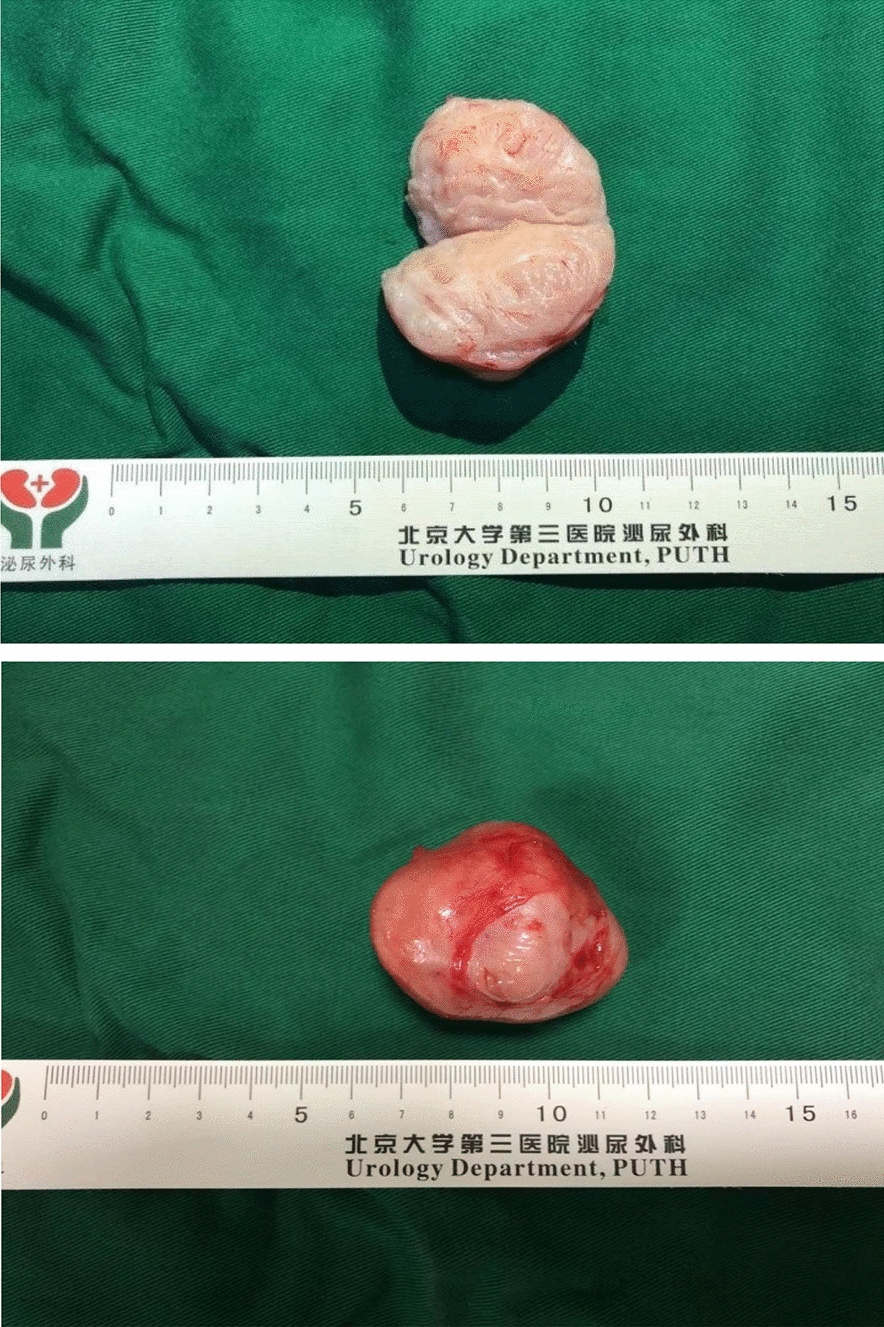
The resected tumor, which was 5.5*4.5*3.8 cm in size

**Fig. 4:**
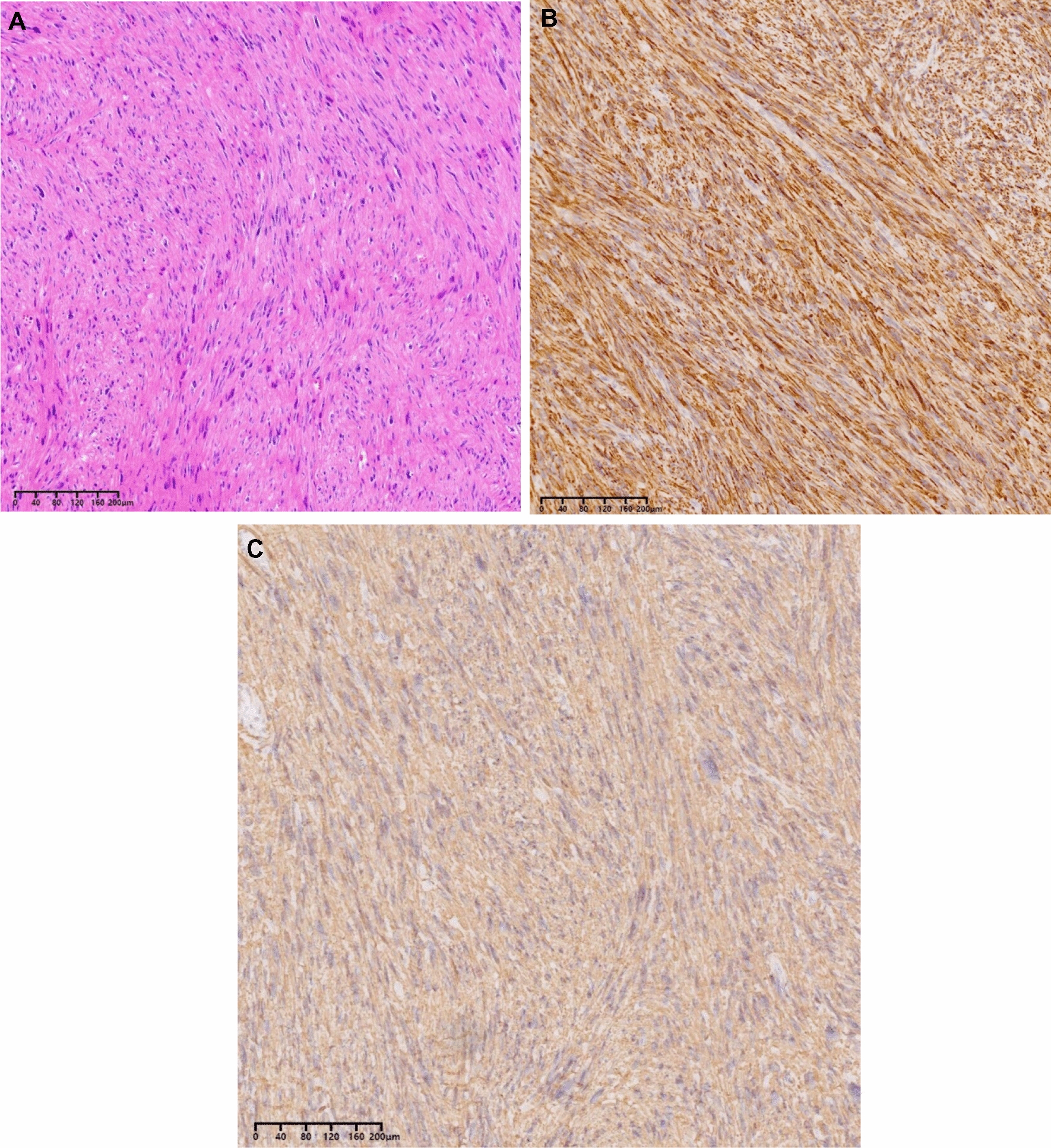
Histological feature of RVLMS. **A**: H&E 10x. 4B and 4C: desmin **B** 10 × and SMA **C** 10x

Total operation time was 224 min, and total blood loss during surgery was 200 ml. The resected tumor was 5.5*4.5*3.8 cm in size (Fig. 3). On the first day after operation, the hemoglobin level was 113 g/L. Postoperative ultrasound showed no obvious free effusion in the abdominal cavity. On the 6th day after the operation, the drainage tube was removed, and the patient was discharged from the hospital. Postoperative pathology results confirmed the renal vein leiomyosarcoma. The tumor was irregular in shape, with negative margins. Pathology revealed spindle cell sarcoma, diffuse severe atypia, S-100 (−), SMA (+), desmin (+), CD34 (−), CD99 (+) (Fig. 4).

**Fig. 5:**
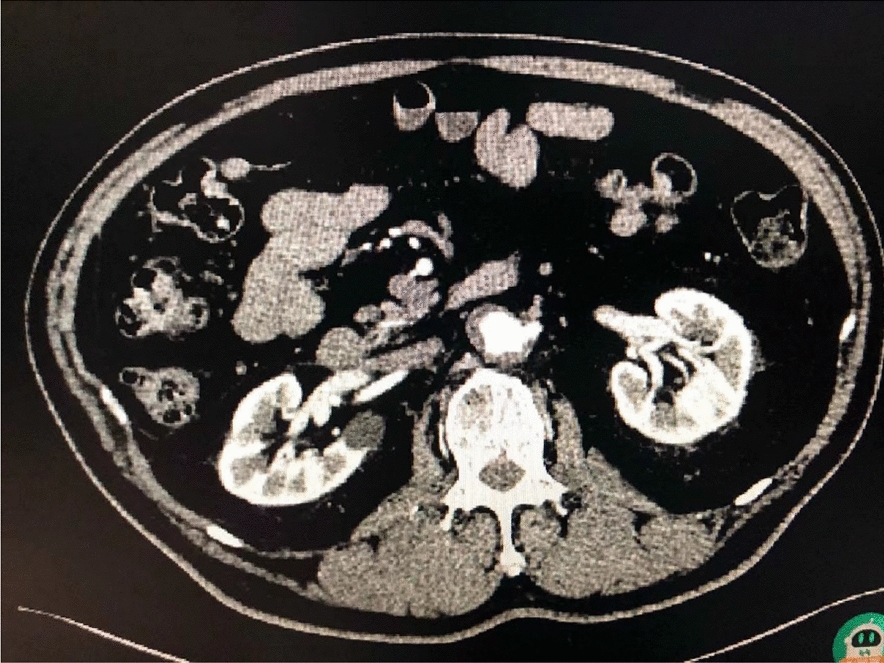
Postoperative abdominal CT shows no sign of local recurrence or distant metastases

At his last follow-up, 27 months after the surgery, the patient is alive without any evidence of local recurrence or distant metastases (Fig. 5).

## Discussion and literature review

The points to discuss are the epidemiological characteristics and diagnostic difficulties of RVLMS, as well as differential diagnoses, use of preoperative core needle biopsy (CNB), treatment strategy, surgical approach choice, and prognostic factors of this disease.

RVLMS is very rare. To date, only 67 cases have been reported in literature, and mostly in the form of a single case report [1]^.^ RVLMS predominantly occurs in women (82%), with a peak incidence in the fifth or sixth decades of life—the tumor is predominantly located on the left side (60%). In terms of clinical manifestations, RVLMS is characterized by abdominal pain (49%), presence of a mass on physical examination (15%), and weight loss (13%) [4].

## Imaging features

Imaging findings are easily confused with, and are difficult to distinguish from those of retroperitoneal tumors and vascular invasion of renal cell carcinoma (RCC). According to Kaushik, the typical CT appearance of a RVLMS is a homogenous, well-circumscribed solid mass with minimal contrast enhancement in the region of the renal hilum [5]. On MRI, RVLMS typically shows up as a well-defined lesion characterized by an isointense signal to the kidney on T1-weighted images and slightly increased signal intensity on T2-weighted images, although less intense in comparison to the kidney [5].

## Differential diagnoses and preoperative CNB

Due to the similarities in the preoperative clinical manifestations, and imaging studies between RVLMS and paraganglioma (PG), a differential diagnosis should be made, especially in patients with a history of hypertension. PG originating from the retroperitoneum or mediastinum is an extra-adrenal pheochromocytoma, which can occur in any part of the abdominal chromaffin tissue, and can be found in the paravertebral ganglion and the bladder [6]. According to literature, the typical CT appearance of a PG is that of a mass greater than 3 cm in diameter, with a round, regular shape, and clear edges. Although the CT manifestations of PG have typical characteristics, they lack specificity . Therefore, it is extremely difficult to diagnose extra-adrenal paraganglioma by the CT alone. PG can be diagnosed by the changes in the level of catecholamines, and their metabolites in the blood [7].

Another disease that needs to be differentiated from RVLMS is retroperitoneal sarcoma (RPS). According to literature, in order to distinguish RPS from other types of retroperitoneal masses, CT-guided core needle biopsy (CNB) is recommended [8]. Wilkinson reported that the preoperative CNB of retroperitoneal sarcoma is safe and will not affect the prognosis of the tumor [9]. However, in cases of suspected paraganglioma, preoperative CNB should be performed with caution, because it may lead to pheochromocytoma crisis. In addition, CNB may lead to potential surgical complications, including renal vein injury and hemorrhage.

## Pathologic features

With regard to the pathological features of RVLMS, they tend to display bundles of spindle-shaped cells, with flat nuclei and fibrillary appearing cytoplasm. RVLMS originating from the retroperitoneum shows nuclear atypia with mitoses. However, it has been reported that the diagnosis cannot be ruled out in patients with inactive mitosis [4]. Immunohistochemistry of LMS usually demonstrates positive staining for myogenic markers such as caldesmon, desmin, or smooth muscle actin (SMA) in greater than 70% of cases, and less frequently stains positive for cytokeratin and epithelial membrane antigen (EMA) in roughly 40% of cases [10]. 

## Treatment strategy

Leiomyosarcomas are highly malignant, and have the potential to metastasize to lymph nodes and distant sites. Therefore, early and complete resection of the tumor can improve the prognosis. Before the operation, thorough examination should be performed, and an MDT meeting should be held to fully analyze and make differential diagnoses.

At present, many surgeons choose radical nephrectomy (RN) over local excision (LE) as the first treatment choice for the following the reasons: (1) owing to the high malignant potential of leiomyosarcoma, cancer cells may invade the inferior vena cava and the kidney. (2) Vascular reconstruction after tumor resection may potentially lead to complications, such as anastomotic leakage, anemia or hemorrhagic shock. (3) It is believed that the possibility of tumor recurrence is greater following tumor resection. However, there is a lack of systematic support regarding the best choice of surgical treatment. We looked at the 67 patients that have been reported in the past. Including our case, we only managed to find surgical approach and follow-up data of 55 cases [1, 2, 4, 5, 8, 11–56]. Among them, 48 patients had a radical nephrectomy (87%), and 7 patients (including our case) had local excision and preserved kidney (13%). Interestingly, from the 48 patients that had undergone RN, 33 were still alive, and 15 were dead. From the 7 patients that underwent LE, 5 patients, including our case were alive [8, 13, 16, 17], and without local recurrence or distant metastases 24 months, 78 months, 8 months, 24 months, and 27 months after the surgery, while the other 2 patients [14, 15] were unfortunately dead (Table 1). Although we believe that RN has advantages over LE in terms of surgical difficulty and complications, for patients with contralateral renal insufficiency, solitary kidney, or a strong desire to preserve the kidney, LE with vascular reconstruction is a feasible option. Kolodziejski et al*.* reported that preservation of the kidney should always be considered when the tumor does not infiltrate the renal hilum [8]. In our case, the patient is still alive, and without evidence of local recurrence or distant metastases 27 months after LE. With regard to the choice of surgery, we believe that further studies with larger sample numbers should be conducted, in order to arrive at a more certain conclusion.

**Table 1:** LMS patients underwent local excision and preserved kidney

Year of publication	Author	Sex	Surgical treatment	Survival	Local recurrence or metastatic spread
1976	Gierson [13]	F	Local excision	78 months NED	No LR, no Mets
1977	Stringer [14]	F	Local excision	72 months DOD	36 months
1982	Dufor [15]	F	Local excision	18 months DOD	No LR, 12 months Mets
2004	Kolodziejski [8]	F	Local excision	24 months NED	No LR, no Mets
2005	Hisa [16]	F	Local excision	8 months NED	No LR, no Mets
2014	Parker M [17]	F	Local excision	24 months NED	No LR, no Mets
2022	Baheen	M	Local excision	27 months NED	No LR, no Mets

Patients underwent local excision (LE)

*NED* no evidence of the diseases, *DOD* died with the diseases. *LR* local recurrence

## Prognostic factors

According to the current literature, the risk of local recurrence and distant metastasis is significantly increased when the tumor is greater than 3 cm in diameter, and when the margins of the resected specimen are positive for tumor cells [4, 55]. Grignon et al*.* reported that the probability of local recurrence after operation is 40%, and that distant metastases can reach the lung, liver, skin and soft tissues [11]. Aguilar et al*.* analyzed 30 cases reported in the literature and found that 30% of patients (average follow-up 78 months) had no local recurrence or distant metastases, 23% had local recurrence and distant metastases but were still alive (average follow-up 48 months), and 37% died following local recurrence [4]. Brandes et al*.* [12] reported that RVLMS exhibits a poor 5-year survival rate. In our case, the patient is alive and without local recurrence or distant metastases 27 months after surgery. Adjuvant therapy and regular follow-up both play important roles in the treatment of RVLMS.

## Limitations

Our study is not without limitations. The number of patients that choose to remove the tumor and preserve the kidney is a lot smaller than that of patients that choose radical nephrectomy. The duration of follow-up in both groups is also inadequate. In addition, the reasons why a particular form of surgical treatment was chosen over another are not mentioned in most cases. Therefore, the best form of surgical treatment remains unknown. However, with our initial results, we believe that the form of surgical treatment may not be the most important factor in determining the OS for these patients. We hope that future studies with larger sample numbers will be carried out, to find out how best these patients can be managed.

## Conclusion

In this study, we shared our experience about the diagnosis and treatment of a patient with RVLMS at our center. RVLMS is very rare, and lacks specific clinical manifestations and features on imaging studies. Most patients are diagnosed intra-operatively or following postoperative pathology. Early and complete resection is considered the first choice of treatment, and whether or not to preserve the kidney depends on the patient's condition. RVLMS is highly malignant and it may recur locally or metastasize to distant locations, therefore, adjuvant therapy and regular follow-up should be carried out regularly after surgery.

## Data Availability

The datasets used and analyzed during the current study are available from the corresponding author on reasonable request.

## Abbreviations

RVLMS: Renal vein leiomyosarcoma
PG: Paraganglioma
RPS: Retroperitoneal sarcoma
RCC: Renal cell carcinoma
SCr: Serum creatinine
VMA: 24Hr 3-methyl-4-hydroxymandelic acid
ACTH: Adrenocorticotropic hormone
RAAS: Renin–angiotensin II–aldosterone system
CNB: Core needle biopsy
